# Isolation and Characterization of a Variant Psedorabies Virus HNXY and Construction of rHNXY-∆*TK*/∆*gE*

**DOI:** 10.3390/ani10101804

**Published:** 2020-10-04

**Authors:** Fengsun Wu, Yujin Lv, Shijun Zhang, Lingling Liu, Yuchen Wu, Pandeng Zhao, Zhifeng Peng, Shengli Liu, Zhonghua Zhang, Wengang Li

**Affiliations:** 1 College of Veterinary Medicine, Henan University of Animal Husbandry and Economy, Zhengzhou 450046, China; 80342@hnuahe.edu.cn (F.W.); 80775@hnuahe.edu.cn (Y.L.); 80399@hnuahe.edu.cn (L.L.); 80110@hnuahe.edu.cn (Y.W.); 80366@hnuahe.edu.cn (P.Z.); zfpeng2006@126.com (Z.P.); 2 Swine Disease Prevention Engineering Research Center of Henan Province, Zhengzhou 450046, China; 3 Department of Animal Science, Henan Agricultural University, Zhengzhou 450046, China; 18134405668@163.com (S.Z.); shengli0529@163.com (S.L.); zhangzhonghua0309@163.com (Z.Z.)

**Keywords:** pseudorabies virus, virus isolation, genetic variation analysis, gene-deletion strain

## Abstract

**Simple Summary:**

Pseudorabies virus (PRV) is a common pathogen in multiple animal species, particularly in pigs, which is now one of the most important factors on pig husbandry loss. This study isolated a PRV variation strain HNXY and performed the etiology characterization of HNXY. Moreover, the attenuated rHNXY-∆*TK*/∆*gE* presented satisfactory safety among susceptible animals, which It is worthy of doing more studies on this variation strain as PRV vaccine candidate.

**Abstract:**

The outbreak of pseudorabies in China, caused by more virulent pseudorabies virus (PRV) than the classical strains, has led to considerable economic losses. In this study, PRV strain HNXY was isolated from the Henan province of China in 2015 from the pig farm with severe reproductive failure in sows and a high mortality in piglets. The 50% tissue culture infectious doses (TCID_50_) of HNXY in Vero cells were examined to be 10^6.5^/mL, and the neutralisation titer against Bartha-K61 was significantly higher than against HNXY when tested with the serum from Bartha-K61 vaccinated pigs. The 50% lethal doses (LD_50_) of HNXY to six-week-old BALB/c mice and two-month-old PRV-free pigs were both 10^2.3^ TCID_50_. HNXY was classified as genotype II, and numerous amino acid variations were found in gB, gE, gC, gD, TK, and RR1 proteins, compared with PRV from other countries or those prevalent in China before 2012. The attenuated rHNXY-∆TK/∆gE was further constructed, which presented significantly smaller plaques than HNXY, as well as the similar growth kinetics. rHNXY-∆*TK*/∆*gE* was confirmed to be non-pathogenic to six-week-old BALB/c mice and zero-day-old piglets. This study isolated updated PRV promising to develop into a new vaccine candidate.

## 1. Introduction

Pseudorabies virus (PRV), also known as *Suid alphaherpesvirus 1* and Aujeszky’s disease virus, belongs to subfamily *Alpha herpesviral* within the family *Herpesviridae*. PRV contains ~145 kb genome and at least 72 genes [1]. It has a broad host range and can infect about 35 species of animals as reported, among which pigs were the primary host and reservoir of PRV [2,3]. PRV has caused great economic losses in the hog industry worldwide due to fatal encephalitis and high mortality in newborn pigs, reproductive failure in sows, and respiratory distress and growth block in growing and fattening pigs.

Although they have only two genotypes based on the gC gene according to the Ye’s study [4], the PRV strains exhibit various biological characteristics, including proliferative properties, virulence, and pathogenicity [5]. Therefore, every country in the world has taken active measures to prevent and control this disease and has been effectively controlled. However, with the deepening of PRV research, it is found that PRV is also prevalent in the wild boar, which poses a severe challenge to PR purification. More studies have found that the presence of PRV virus in wild pig fetus, which may have another route of transmission [6,7,8,9,10]. There have been many reports of pseudorabies virus showing signs of human infection [11,12]. Recently, it has been reported that a PRV strain was isolated from an acute human encephalitis case [13].

After the first report of pseudorabies cases in China in 1948, there was a pandemic in the 1980s. Due to the widespread use of efficacious attenuated vaccines with the key virulence genes (such as *gE*) knocked out, including the famous Bartha-K61 vaccine, PR was gradually controlled in China. However, since late 2011, PR has broken out in a large number of pig farms even in those where pigs had been routinely vaccinated with Bartha-K61. The positive rate of *gE* antibody after 2011 in the pig serum samples was much higher than that before 2011 [14,15]. It has been reported that the virus has changed both in gene sequence and virulence [14,16,17]. What’s more, the Bartha-K61 vaccine was reported to be unable to provide complete protection against the variant PRV strains in many studies [14,18]. Currently, several attenuated PRV strains have been constructed and confirmed to be safe and able to induce immune protection in response to a parental PRV challenge, including rPRVTJ-delgE/gI/TK [19], JS-2012-△gE/gI [20], rPRVXJ-delgI/gE-EGFP [21], rSMX△gI/gE△TK [22], the triple gE/gI/TK gene-inactivated HeN1 PRV strain [23], and the gE gene-deleted PRV based on the PRV HN1201 [24]. However, since HeN1, SMX, HN1201, and HNX strains isolated in 2012 and their respective attenuated strains were reported [22,23,25,26], there have been no reports about variant strains for vaccine candidates from Henan province.

In this study, a pig farm in Xinyang City Henan province suffered from PRV infection in 2015. Although Bartha-K61 had been vaccinated every year in this farm, a high proportion of the sows were found to have reproductive failure such as abortions, reduced live and healthy litter size, and more piglets had such symptoms as high fever, anorexia, respiratory distress, and neurological symptoms. The pathogen PRV strain, named HNXY, was isolated from the tissues of the dead piglets, and then was plaque-purified. Several antigen or virulence-related genes and the basic biological characteristics of HNXY were analyzed. Moreover, the double gene deletion strain rHNXY-∆*TK*/∆*gE* was constructed and confirmed to be 100% safe to SPF mice and newborn piglets.

## 2. Materials and Methods

### 2.1. Cells, Viruses and Plasmids

Vero cells, MDBK cells, and HEK293 cells were grown in Dulbecco’s modified Eagle’s high glucose medium (DMEM) supplemented with 10% fetal bovine serum (FBS) (GIBCO, Waltham, MA, USA) at 37 °C in 5% CO_2_. Homogenized PRV *gE* positive tissues. Bartha-K61 vaccine strain was bought from Wuhan *keqian* Biology Co., Ltd. (Wuhan, China). Plasmid pSIMPLE-19 EcoR V/BAP (TaKaRa, Tokyo, Japan) was used as a vector linking genes and was sequenced in BGI (Beijing, China). The pUC19, pCDNA3.1-EGFP, and pCDNA3.1-Cre (Fenghbio, China) were applied for recombinant plasmid construction and for homologous reorganization [20].

### 2.2. Virus Isolation and Purification

Homogenized PRV *gE* positive tissues were frozen-thawed for 3 times and then centrifuged at 10,000 r/min at 4 °C for 10 min. The supernatants were collected for following virus isolation and purification with Vero cells as previously described [1].

### 2.3. Neutralizing Test

Anti-serum of Bartha-K61 vaccinated pigs were obtained and confirmed to be gB antibody positive while gE antibody negative by ELISA (IDEXX, Westbrook, ME, USA). The neutralizing titers of the serum samples to HNXY and Bartha-K61 were determined on Vero cells as described before [27].

### 2.4. PCR Amplification of Full-Length gB, gE, gC, gD, TK, RR1 and RR2 of HNXY and Phylogenetic Analysis

Viral genomic DNA of HNXY was extracted with QIAamp DNA Blood Mini Kit (QIAGEN, Hilden, Germany) following the instructions. The full-length *gB*, *gE*, *gC*, *gD*, *TK*, *RR1* and *RR2* genes were amplified with PrimeSTAR and GC buffer (Takara, Japan) in a 50μL reaction using the following primers designed based on PRV DL14/08 strain (Genbank: KU360259) by Primer Premier 5. 0: *gB*-F 5′-ATGCCCGCTGGTGGCGGTCTTTGG-3′, *gB*-R 5′-CTAGGGGGCGTCGGGGTCCTCGTTC-3′; *gE*-F 5′-ATGCGGCCCTTTCTGCTGCGCG-3′, *gE*-R 5′-TTAAGCGGGGCGGGACATCAACA-3′; *gC*-F 5′-ATGGCCTCGCTCGCGCGTGCGATGC-3′, *gC*-R 5′-TCACAGCGCGGACCGGCGGTAGTAG-3′; *gD*-F 5′-ATGCTGCTCGCAGCGCTATTGGC-3′, *gD*-R 5′-CTACGGACCGGGCTGCGCTTTTA-3′; *TK*-F 5′-ATGCGCATCCTCCGGATCTACCTCG-3′, *TK*-R 5′-TCACACCCCCATCTCCGACGT-3′; *RR1*-F 5′-ATGGCCTCCCCCGTCGTGCCCGC-3′, *RR1*-R 5′-TCACAGGTGGCAGCTCGTGCAGA-3′; *RR2*-F 5′-ATGGAGTACTTTTACACGTCCC-3′, *RR2*-R 5′-CTACAGGTCGTTCACGACGGTCCCC-3′. The PCR was conducted for 30 cycles at 98 °C for 10 s, 68 °C for several minutes (1 kb/min, determined by the length of the genes). The PCR products were cloned into pSIMPLE-19 EcoR V/BAP plasmid and sequenced by BGI (Beijing, China). Phylogenetic analysis was performed according to the reference sequences of PRV from GenBank with MEGA software (version 6.0) (Arizona State University, Tempe, AZ, USA) by the Clustal W method with 10 00 bootstrap replicates [17].

### 2.5. One-Step Growth Analysis

One-step growth kinetics test was conducted as previously described [28] with some modifications. Vero cells were infected with HNXY or rHNXY-∆*TK*/∆*gE* at a multiplicity of infection (MOI) of 0.05. And cells and supernatant were both harvested at the 12th, 24th, 36th, 48th, 60th, 72th, 84th and 96th hour after penetration at 37 °C. The samples were frozen-thawed for 3 times and titers of virus were determined by 50% tissue culture infectious doses (TCID_50_) in Vero cells.

### 2.6. Construction of Transfer Vectors

The 1.2 kb upstream and downstream homologous fragments flanking the PRV *TK* or *gE* genes were amplified by PCR using primers listed in Table 1. Fragments were ligated together at *spel* I site, and inserted into *EcoR* I and *Hind* III sites of the pUC19 vector to create pUC19-*TK*-U-D or pUC19-*gE*-U-D (Figure 4A). Then the EGFP expression cassette, containing CMV promoter and polyA terminator, was amplified with primers loxP-EGFP-F and loxP-EGFP-R (Table 1) and was cloned into the pUC19-*TK*-U-D or pUC19-*gE*-U-D at *spel* I sites to construct pUC19-*TK*-U-D-EGFP or pUC19-*gE*-U-D-EGFP vector (Figure 4A).

### 2.7. Generation of rHNXY-∆TK/∆gE

The *gE* and *TK* genes were deleted sequentially by homologous reorganization [22,24]. Briefly, HEK293 cells were co-transfected with 500 ng pUC19-*gE*-U-D-EGFP cassette and 2 μg PRV genome with Lipofectamine 2000. When cytopathic effects (CPE) were observed, the culture was harvested and plaque purified in Vero cells for 3~5 times as described before [1], thus generating virus rHNXY-∆*gE*-EGFP with green fluorescence. To remove the EGFP expression cassette, rHNXY-∆*gE*-EGFP (MOI = 1) was inoculated to HEK293 cells 6 h after 2 μg pCDNA3.1-Cre plasmids transfection. And the single plaque without green fluorescence was picked out during the virus purification to obtain rHNXY-∆*gE*. Based on rHNXY-∆*gE*, *TK* gene was knocked out in the same way. PRV genome arrangement and the loci of ∆*TK* and ∆*gE* in rHNXY-∆*TK*/∆*gE* were presented in Figure 4B. The gene deletion in purified recombinant viruses was determined by PCR with primers presented in Table 1.

### 2.8. Animal Experiment

The current study was carried out by the guidelines established by the China Regulations for the Administration of Affairs Concerning Experimental Animals (1988). All procedures and handling techniques were approved by The Institutional Animal Care and Use Committee (IACUC) of Henan University of Animal Husbandry and Economy (Use Committee of Henan University of Animal Husbandry and Economy; China; Permit number: Animal Welfare Assurance No. HNUAHE-2018-003). All efforts were made to provide the ethical treatment and minimize suffering of animals employed in this study.

#### 2.8.1. Experimental Infection of Mice

To determine the pathogenicity of HNXY and Bartha-K61, 110 6-week-old specific-pathogen-free (SPF) BALB/c mice were randomly divided into 11 groups with 10 mice in each group. Mice in groups 1–5 were injected subcutaneously (s.c.) with 100 μL HNXY of different doses (10^1^–10^5^ TCID_50_), respectively, and groups 6–10 were inoculated with Bartha-K61 in the same manner. Group 11 was the control injected with DMEM. The observation lasted for 14 days.

To verify the safety of rHNXY-∆*TK*/∆*gE*, thirty 6-week-old SPF BALB/c mice were randomly divided into 3 groups with 10 mice in each group. Group 1 and 2 were inoculated s.c. with 100 μL (10^5^ TCID_50_) of HNXY and rHNXY-∆*TK*/∆*gE*, respectively. Group 3 was the uninfected control with DMEM. The observation lasted for 14 days.

#### 2.8.2. Experimental Infection of Pigs

To investigate the viral pathogenicity, thirty 2-month-old PRV-free pigs were randomly divided into 6 groups with 5 pigs in each group. Group 1–5 were challenged with HNXY in an intranasal route at different doses (10^1^–10^5^ TCID_50_) and the remaining group with DMEM as control. The observation lasted for 25 days.

To check the safety of rHNXY-∆*TK*/∆*gE*, twenty-four 0-day-old newborn piglets were randomly divided into 3 groups. Group 1 and 2 were inoculated with rHNXY-∆*TK*/∆*gE* at a dose of 10^5^ TCID_50_, respectively through nasal and intramuscular infection. Group 3 was the control with DMEM. Blood samples were collected at the 25th day post inoculation (dpi) for antibody detection by ELISA. The observation lasted for 25 days.

### 2.9. Accession Numbers

The sequences obtained in the current study were submitted to GenBank (National Center for Biotechnology Information, Bethesda, MA, USA) and are available under the accession numbers from MN003371 to MN003377.

### 2.10. Statistical Analyses

Data are expressed as the mean ± standard deviation (mean ± SD). Statistical significance of the differences between each group was analyzed by a one-way analysis of variance (ANOVA) or two-way ANOVA embedded in GraphPad Prism, version 6.0 (GraphPad Software Inc., La Jolla, CA, USA). Differences between the in vitro bacterial invasion rates were determined by Student’s *t*-test. *p* < 0.05 (*) was considered significant, and *p* < 0.01 (**) was considered extremely significant.

## 3. Results

### 3.1. Virus Isolation

**Figure 1:**
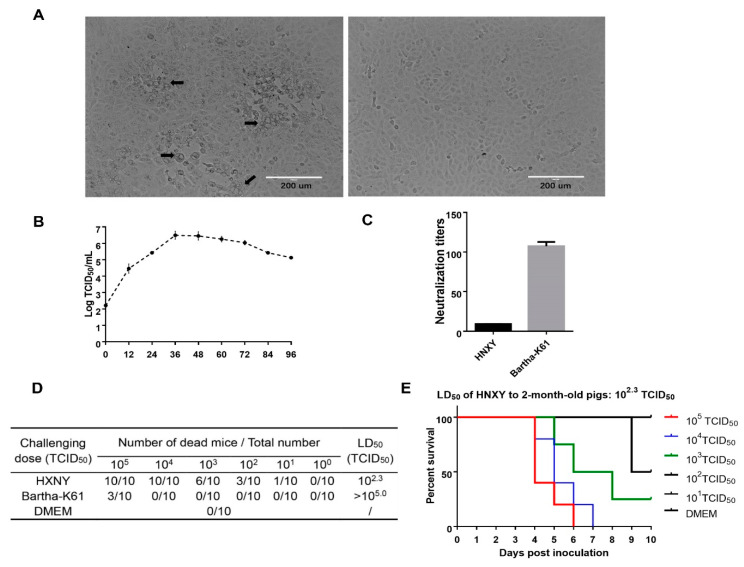
Proliferative characteristics of HNXY. (**A**) Cytopathic effect caused by HNXY on Vero cells. Arrows mark the reticulated cells and large syncytia. (**B**) One-step growth curve of HNXY on Vero cells. (**C**) Neutralization test of HNXY and Bartha-K61 with antisera of Bartha-k61 vaccinated pigs. (**D**) The number of dead mice in groups challenged with HNXY, Bartha-K61, and DMEM, respectively. (**E**) The survival curve of 2-month-old pigs challenge with HNXY at various doses. 5 pigs were arranged in each group.

HNXY was isolated from the gE positive tissues after proliferation and three-time purification. Typical CPE characterized by reticulated cells (swelling and rounding cells) and large syncytia was observed in the HNXY infected Vero cells (Figure 1A). PCR or reverse transcription PCR showed that the cell cultures were PRV positive but were negative for other important swine pathogens (Figure S1).

Several properties about proliferation and virulence of HNXY were investigated. The virus proliferated to the highest titer (10^6.5^ TCID_50_/mL) at the 36th-h post inoculation (HPI) when the Vero cells were infected with a dose of 0.05 MOI, while the infectious virions gradually decreased to 10^5.0^ TCID_50_/mL in the following 60 h. (Figure 1B). When tested with the serum from Bartha-K61 vaccinated pigs, the neutralization titer of Bartha-K61 with a mean of 1:107 was significantly higher than that of HNXY with a mean of 1:9.08, indicating insufficient protection of Bartha-K61 against HNXY (Figure 1C). After challenged with Bartha-K61 and HNXY, the mice exhibited various symptoms and mortality. Compared with the Bartha-K61 infection causing the death of only 3 mice challenged with 10^5.0^ TCID_50_, the HNXY infection at the dose of 10^5.0^ and 10^4.0^ TCID_50_ resulted in 100% acute death, pruritus, and crazy scratching and biting at the injection site (Figure 1D). The LD_50_ of HNXY and Bartha-K61 to the mice was 10^2.3^ TCID_50_ and >10^5.0^ TCID_50_, respectively (Figure 1D). All the pigs infected with HNXY by nasal delivery suffered from elevated body temperature since 30HPI and the peak of the death occurred during the 4th- to 7th-day post infection. The LD_50_ of HNXY to the pigs was ultimately determined as 10^2.3^ TCID_50_ (Figure 1F).

### 3.2. Genetic Variation Analysis of gB, gE, gC, gD, TK, RR1 and RR2 Proteins

**Figure 2:**
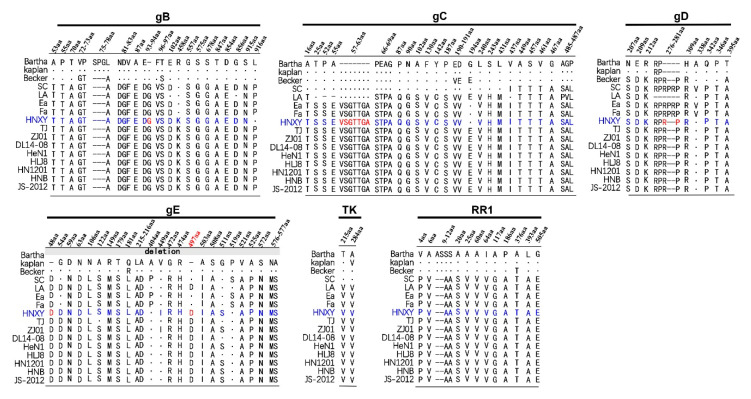
Genetic variation analysis. The variations in amino acid sequences of gB, gC, gD, gE, TK, and RR1 proteins were collected in the lists, respectively. Sequences in Bartha strain or Kaplan (gE protein) were taken as the reference strain. Amino acids (AA) in red indicate insertions and those in blue indicate mutations.

Compared with those in PRV strains from China and other countries, gB, gE, gC, gD, TK, RR1, and RR2 proteins in HNXY displayed regular insertions, deletions, or substitutions in their amino acid (AA) sequences, except for RR2 (Figure 2). The specific differences of each protein are described as follows: (1) In gB of HNXY, there were 22 discontinuous substitutions at site 53 (A→T), 55 (P→T), 70 (T→A), 72 (V→G), 73 (P→T), 78 (L→A), 81 (N→D), 82 (D→G), 83 (V→F), 87 (A→E), 93 (E→D), 96 (F→V), 97 (T→S), 102 (E→D), 458 (R→K), 557 (G→S), 575 (S→G), 678 (S→G), 847 (T→A), 854 (D→E), 856 (G→D), and 915 (S→N), three continuous AA deletions (S, P and G) at site 75–77, and one insertion of G at site 94. Most of the above changes were consistent with those observed in Chinese PRV reference strains, but R458K mutation was exclusively found in PRV isolated after 2012 in China. In addition, the amino acid leucine at site 916 of HNXY was distinct from proline in other Chinese strains. (2) The gC analysis showed that 27 AA mutations and seven uninterrupted AA insertions (VSGTTGA at site 57 to 63) occurred in PRV isolated after 2012 in China, including HNXY. However, at site 194, glycine in HNXY, as well as in Bartha, Kaplan, Ea, and Fa, differed from glutamate in Chinese novel strains and Becker. (3) The gD analysis indicated seven mutations at various sites and two insertions at site 278 and 281 in HNXY and other Chinese isolates, except for LA. Besides, one additional mutation of A338V and two additional insertions at site 279 and 280 were observed in SC, Ea, and Fa. (4) The gE analysis revealed that HNXY had 20 mutations and two insertions of aspartate at site 48 and 497, compared with Kaplan and Becker. Among these differences, the mutation of G54D and the insertion of aspartate at position 497 were shared by all the Chinese strains after 2012, while A122S, V449I, and G511S were common to partial novel strains. Besides, the mutations of A404P and P519S were shared by Ea and Fa rather than by the Chinese novel strains. (5) The relatively conserved TK showed only two mutations of T215V and A284V, which were shared by all Chinese reference strains. (6) The two AA deletions and 13 mutations were observed in RR1 in all Chinese strains including HNXY.

As illustrated in Table 2, the minimum amino acid sequence homologies for gB, gC, gD, gE, TK, RR1, and RR2 proteins between HNXY from China and PRV strains from other countries were 96.4, 91.7, 96.0, 95.5, 99.1, 96.8, and 97.4%, respectively. The minimum amino acid sequence homologies for the seven proteins between HNXY in 2015 and the strains prevalent in China before 2012 were 98.7, 94.0, 98.3, 98.8, 99.1, 99.2 and 99.0%. When HNXY in 2015 was compared with the Chinese isolates after 2012, the homology for gC and gD were 98.6 and 98.3%, while all other proteins have more than 99% homology.

### 3.3. Phylogenetic Tree Analysis

**Figure 3:**
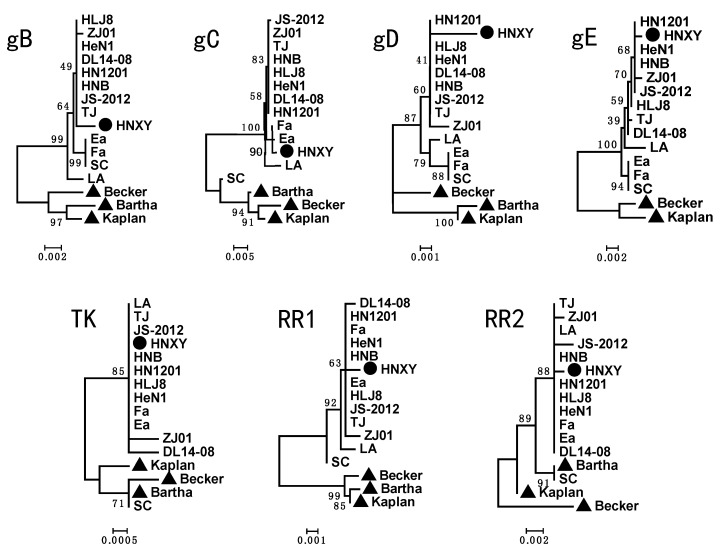
Phylogenetic trees based on gB, gC, gD, gE, TK, RR1 and RR2 genes, respectively. “●” is HNXY, the PRV isolates from Henan, “▲” is the PRV isolates from other countries.

The phylogenetic trees based on all the analyzed genes showed that HNXY fell into the same cluster with the PRV isolates from China while it was far away from the strains from other countries, suggesting HNXY belongs to genotype II (Figure 3). The phylogenetic trees based on the *gB*, *gD*, *gE*, *TK*, *RR1* and *RR2* genes exhibited that HNXY had a close relationship with the PRV isolated after 2012 from China. However, HNXY exhibited a close relationship to PRV Ea and Fa isolated before 2012 in the tree based on *gC* gene. Moreover, HNXY was located right next to the strains HeN1, HN1201, and HNB isolated from Henan province in 2012 in the trees based on most genes, but HNXY was relatively far away from these strains when trees were constructed based on *gB* and *gC*.

### 3.4. Generation of rHNXY-∆TK/∆gE

**Figure 4:**
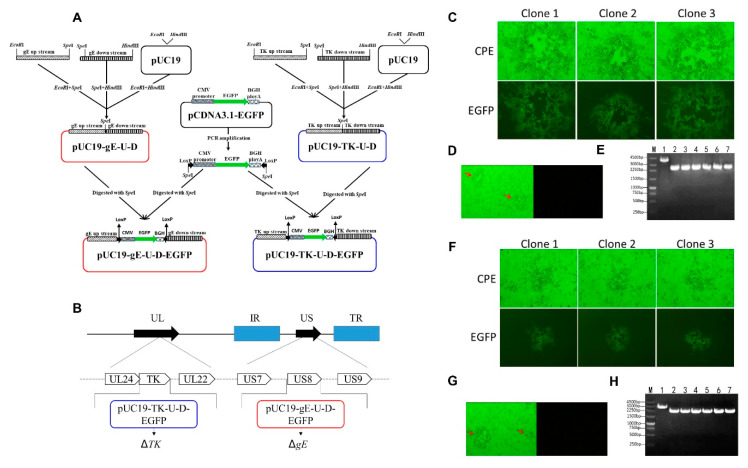
Construction and identification of HNXY-∆TK/∆gE. (**A**) The diagram of the construction of the transfer vectors. (**B**) PRV genome arrangement and the loci of ∆TK and ∆gE in rHNXY-∆TK/∆gE. (**C**) rHNXY-∆gE-EGFP with green fluorescence under the fluorescent microscope. (**D**) rHNXY-∆gE-EGFP virus without green fluorescence under the fluorescent microscope. (**E**) PCR identification of gE deletion. Line 1 was HNXY, line 2, 3, 4, 5, 6, and 7 were the randomly selected plaques in the last round of purification of rHNXY-∆gE. (**F**) rHNXY-∆TK/∆gE-EGFP with green fluorescence under the fluorescent microscope. (**G**) rHNXY-∆TK/∆gE virus without green fluorescence under the fluorescent microscope. (**H**) PCR identification of TK deletion. Line 1 was rHNXY-∆gE, line 2, 3, 4, 5, 6, and 7 were the randomly selected plaques in the last round of purification of rHNXY-∆TK/∆gE. “Red Arrows” of 4D is plaques without green fluorescence were screened from the EGFP positive plaques during the purification. “Red Arrows” of 4G is rHNXY-∆TK/∆gE virus without green fluorescence under the fluorescent microscope.

In order to delete *gE* gene from the genome of HNXY, the transfer vector pUC19-*gE*-U-D-EGFP was constructed and was co-transfected with genomic DNA of HNXY into HEK293 cells, thus producing the recombinant virus rHNXY-∆*gE*-EGFP and further purifying the virus though plaque purification based on the expression of EGFP (Figure 4C). Then, the Cre/loxP system was applied to obtain rHNXY-∆*gE* without EGFP. The pCDNA3.1-Cre transfected HEK293 cells were infected by rHNXY-∆*gE*-EGFP. Subsequently, plaques without green fluorescence were screened from the EGFP positive plaques during the purification (Figure 4D). In the last round of purification of rHNXY-∆gE, the 6 randomly picked plaques were identified by PCR to be about 1800 bp shorter than HNXY (Figure 4E), which was consistent with the deletion fragments of *gE* gene as expected.

Based on rHNXY-∆*gE*, *TK* gene deletion was conducted. The EGPF positive plaques were pipetted out of those without green fluorescence to distinguish rHNXY-∆*TK*/∆gE-EGFP from rHNXY-∆*gE*, (Figure 4F). In order to generate rHNXY-∆TK/∆gE, plaque purification was conducted for several times after the recombination under the Cre/loxP system to eliminate viruses expressing EGFP (Figure 4G). Finally, PCR confirmed the *TK* gene deletion according to an 800 bp decrease in the the plaques compared with rHNXY-∆*gE* (Figure 4H).

### 3.5. Growth Properties of rHNXY-∆TK/∆gE and HNXY In Vitro

**Figure 5:**
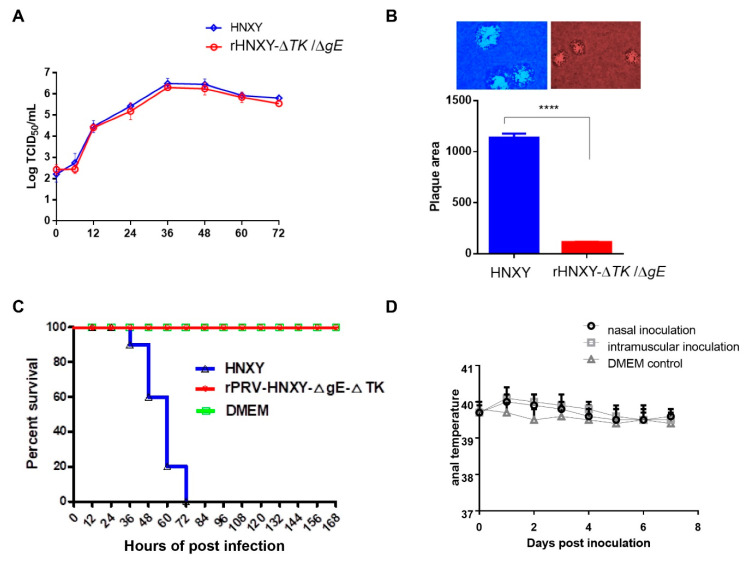
Growth characteristics and safety of HNXY-∆*TK*/∆*gE*. (**A**) One step growth analysis of HNXY-∆*TK*/∆*gE* and HNXY. (**B**) rHNXY-∆TK/∆gE formed significantly smaller plaques than HNXY. **** rHNXY-∆TK/∆gE formed significantly smaller plaques than HNXY. (**C**) The survival of the mice infected with rHNXY-△*TK*/△*gE* and HNXY in 10^5^ TCID_50_. (**D**) The anal temperatures of 0-day-old pigs inoculated with rHNXY-△TK/△gE by nasal or intramuscular during the observation.

The in vitro growth properties of attenuated rHNXY-∆TK/∆gE and wild-type HNXY were compared by one-step growth analysis in Vero cells and by plaque area measurement in MDBK cells. The growth kinetics of rHNXY-∆TK/∆gE were similar to those of HNXY (Figure 5A). While, rHNXY-∆TK/∆gE formed significantly smaller plaques than HNXY (Figure 5B).

### 3.6. Pathogenicity of rHNXY-∆TK/∆gE and HNXY In Vivo

The rHNXY-∆*TK*/∆*gE* was confirmed to be non-pathogenic to mice. All the mice injected with rHNXY-∆*TK*/∆*gE* at a dose of 10^5^ TCID_50_ survived (100%) with no symptoms throughout the observation. However, the mice infected with HNXY had a mortality of 80% at 60 hpi and 100% at 72 hpi, displaying typical pruritus of PRV infection (Figure 5C).

Furthermore, in order to investigate whether rHNXY-∆*TK*/∆*gE* was avirulent to pigs, the newborn piglets were inoculated by conventional vaccinating routes including nasal and intramuscular approaches. As shown in Figure 5D, 0-day-old pigs vaccinated with rHNXY-∆*TK*/∆*gE* by above two routes showed normal anal temperatures after experienced a slight increase during the observation, without clinical symptoms and side effects. The slightly increased anal temperatures may be contributed by inoculation stimulation. In addition, gB antibodies were detected to be positive in all vaccinated groups and negative in the control group at 25 dpi, while gE antibodies was negative in all pigs (data not shown). The results above suggested the safety of rHNXY-∆*TK*/∆*gE* to the newborn piglets.

## 4. Discussion

PRV has been one of the main pathogens causing considerable economic losses in the pig industry since it was first reported in 1947 in China. The application of differentiation of infected from vaccinated animals (DIVA) based on the development of PRV genetic marker vaccines and the companion diagnostic methods [29] greatly reduced the morbidity and mortality. However, the outbreak of PR in China in late 2011 resulted in a sharp increase in gE-antibody positive rate to more than 50% [20]. Moreover, It has been reported that central China including Henan province suffered from a relatively more severe PRV infection than most other parts of China [30,31], and that the gE-antibody positive rates of pig farms and serum samples in Henan Province were 70.16% and 40.08% from January 2011 to May 2013, respectively, ranking the highest compared with those in the other three neighboring provinces [32]. Therefore, it is important to continuously investigate the epidemic of PRV in Henan province.

So far, HeN1, SMX, HN1201, and HNX have been isolated from Henan in 2012, and the respective gene-deletion attenuated strains displaying complete protection against PRV challenge have been constructed [22,23,25,26]. In this study, we isolated and purified the PRV HNXY from a pig farm located in Henan province in 2015. HNXY had a LD_50_ of 10^2.3^ TCID_50_ to 6-week-old BALB/c mice and two-month-old pigs (Figure 1D,E), exhibiting similar pathogenicity with PRV TJ in mice reported in a previous study [16]. Barth-K61 was reported to provide sufficient protection against challenge with the Chinese classical PRV strain SC, but it was not effective enough against the variant strains HeN1 and TJ [14,16]. In addition, the neutralization titer of antisera induced by Bartha-K61vaccine against Bartha-K61 was 11.78 folds higher than that against HNXY (Figure 1C), and it was 2–4 folds higher than that against HeN1 and ZJ01 [14,33], suggesting the differences in the neutralizing epitopes and immunogenicity between Bartha-K61 and HNXY.

Of 11 identified envelope glycoproteins of PRV, gB, gC, and gD could induce strong antibody response and protective immune responses in susceptible animals [34,35,36]. The gE, TK, RR1 and RR2 were reported to contribute greatly to the virulence of PRV [37,38]. The numerous variations in the immunogenic and virulent proteins between the PRV strains from other countries and HNXY from China could provide an explanation to the significant decreased neutralization and protection capacity of Bartha-K61 vaccine to the variant strains. Compared with the Chinese reference strains, HNXY possesses the most similarity in the sequences to the PRV strains prevalent after 2012 and shares 2 aspartic acid insertions at position 48 and 497 in gE protein with these PRV strains, indicating that HNXY is a variant PRV isolate [14]. However, HNXY also shows some specificities, including the sharing of G at site 194 in gC with SC, Ea and Fa, rather than with the variant strains, and the exclusive arrangements of L at site 916 in gB and I at site 449 in gE, as compared to other Chinese strains (Figure 2). In addition, gD is the key glycoprotein on the virion surface to initiate the virus infection [39]. The amino acids at site 279, 280 and 338 in the variant PRV are different from those in the classical PRV, suggesting the conditions for PRV entry into the host cells may have changed. According to a previous study, HNXY belongs to genotype Ⅱ based on *gC* gene analysis [4]. Our analysis of other six genes also illustrates the same genotyping results. HNXY is a variant strain, while it has a closer evolutionary relationship with Chinese classical PRV than with variant strains in *gC* phylogenetic tree, implying the possible recombination within the genome of HNXY (Figure 3). Importantly, in *gB* and *gC* phylogenetic trees, HNXY isolated from Henan province in 2015 was different from PRV strains isolated from same place in 2012, indicating the variations in the isolates from the same geographical area and the necessity of monitoring. Future study could focus on the analysis based on the whole genome to confirm the variations in the sequences and the corresponding changes in functions.

The *gE* and *TK* genes have been known as the excellent targets for PRV attenuated live vaccine since they are crucial for virulence, but not necessary for viral replication [40]. The *gE* deletion will make the application of DIVA possible, but it may result in insufficient safety to newborn piglets [41], therefore, we constructed a double gene-deleted rHNXY-∆*TK*/∆*gE*. BAC techniques, homologous reorganization assays, and CRISPR/Cas9 system are applied to generate gene-deletion strains for final purpose of developing PRV live vaccines by genetic engineering [15,22,23]. In this study, homologous DNA recombination coupled with Cre/Lox system was used to delete *gE* and *TK* genes, reducing the workloads in plaque purification. The rescued viruses form smaller plaques, but they have similar growth features as compared with their parent strains, they can reach 106.5 TCID_50_/_mL_ within 36 h. It is proved that *gE* and *TK* genes are non-essential genes for proliferation, and the deletion does not affect the proliferation of double gene-deleted rHNXY-∆*TK*/∆*gE* on BHK-21 cells.

In the safety test, all the mice were no symptoms throughout the observation when injected with rHNXY-∆*TK*/∆*gE* at a dose of 10^5^ TCID_50_, while the piglets injected with the female parent strain PRV-HNXY at a dose of 10^5^ TCID_50_ had a lethal rate of 100%. In addition, the piglets were injected with the rHNXY-∆TK/∆gE through both nasal drops and intramuscular injections, and no clinical symptoms and side effects were observed. The results showed that the pathogenicity of the rHNXY-∆*TK*/∆*gE* to mice and piglets decreased significantly. The results showed that the double gene-deleted rHNXY-∆*TK*/∆*gE* reduced the pathogenicity of mice and piglets significantly, while the safety of the double gene-deleted rHNXY-∆*TK*/∆*gE* in sheep and its protection against the prevalent PRV remain to be further verified.

## 5. Conclusions

In this study, a PRV variation strain HNXY was isolated and purified, presented high pathogenicity to mice and piglets. The HNXY belonged to genotype II, and the following genetic variation analysis demonstrated multiple insertion or deletion mutation in gB, gE, gC, gD, TK, RR1, and RR2 proteins of HNXY, compared with PRV strains from China and other countries. Further, attenuated rHNXY-∆TK/∆gE was constructed and was confirmed to be non-pathogenic to 6-week-old BALB/c mice and 0-day-old piglets. These results suggested the strain HNXY to be a PRV vaccine candidate.

## Figures and Tables

**Table 1:** Sequences of oligonucleotides used in PCR for construction of rPRV-HeNXY/2015-∆*TK*/∆*gE*.

Primer	Sequence	Details
gE-U-F	5-ccg*gaattc*cctcctcgccgccctgaccctg-3	upstream homologous fragments flanking gE gene
gE-U-R	5-gg*actagt*gacggagataaaacgccaccca-3	
gE-D-F	5-gg*actagt*ataccgggagaaccggt-3	downstream homologous fragments flanking gE gene
gE-D-R	5-ccc*aagctt*aggagcggttgtgga-3	
TK-U-F	5-ccg*gaattc*ccggttgcccacgtacag-3	upstream homologous fragments flanking TK gene
TK-U-R	5-gg*actagt*cccggcgcgcttccgg-3	
TK-D-F	5-gg*actagt*ccctcgcccctcccaccc-3	downstream homologous fragments flanking TK gene
TK-D-R	5-ccc*aagctt*ccgggtcctcgccgaa-3	
loxP-EGFP-F	5-gg*actagt*ataacttcgtatagcatacattatacgaagttattagttattaatagta-3	EGFP expression cassette
loxP-EGFP-R	5-gg*actagt*ataacttcgtataatgtatgctatacgaagttatagccatagagcccac-3	
gE-U-L	5-agcccggtccgtagcctccgcagtac-3	gE deletion verification
gE-D-R	5-cctccgtccactcgccggcgt-3	
TK-U-L	5-ggtgcaccaggtgcaggcacag-3	TK deletion verification
TK-D-R	5-ggcgacggtcgcccgcgcgagg-3	

Italicized nucleotide sequences are corresponding restriction enzyme sites, the underlined sequences indicate the LoxP and the sequences in the box are the 8 bp sequence in the LoxP that determines the recombination pattern.

**Table 2:** Sequence comparisons between HNXY and other PRV strains.

Gene	Compared with Bartha, Kaplan and Becker		Compared with SC, LA, Ea, Fa		Compared with PRV Isolated after 2012 in China	
	Nucleotide Sequence Homology	Amino Acid Sequence Homology	Nucleotide Sequence Homology	Amino Acid Sequence Homology	Nucleotide Sequence Homology	Amino Acid Sequence Homology
*gB*	98.2%–98.4%	96.4%–97.2%	99.5%–99.6%	98.7%–98.9%	99.7%–99.8%	99.3%–99.9%
*gC*	95.9%–96.2%	91.7%–92.5%	96.9%–99.9%	94.0%–99.4%	99.5%–99.6%	98.6%–98.8%
*gD*	98.5%–98.8%	96.0%–97.5%	99.2%–99.4%	98.3%–98.5%	99.4%–99.6%	98.3%–98.5%
*gE*	97.6%–97.8%	95.5%–95.7%	99.4%–99.5%	98.8%–99.3%	99.7%–99.9%	99.0%–99.7%
*TK*	99.6%–99.7%	99.1%–99.4%	99.7%–100%	99.1%–99.7%	99.9%–100%	99.4%–99.7%
*RR1*	98.6%	96.8%–97.1%	99.7%–99.9%	99.2%–99.5%	99.7%–99.9%	99.2%–99.5%
*RR2*	98.4%–99.5%	97.4%–99.0%	99.5%–99.9%	99.0%–99.3%	99.7%–99.9%	99.0%–99.3%

## Supplementary Materials

The following are available online at https://www.mdpi.com/2076-2615/10/10/1804/s1, Figure S1: Identification of the cell cultures.
