# Supplementary material for: Isolation and Characterization of a Variant Psedorabies Virus HNXY and Construction of rHNXY-∆TK/∆gE
Source: Animals (Basel). 2020 Oct 4;10(10):1804. doi: 10.3390/ani10101804 (PMC7600349; doi:10.3390/ani10101804)
Supplement: Supplementary file 1 [file animals-10-01804-s001.pdf]

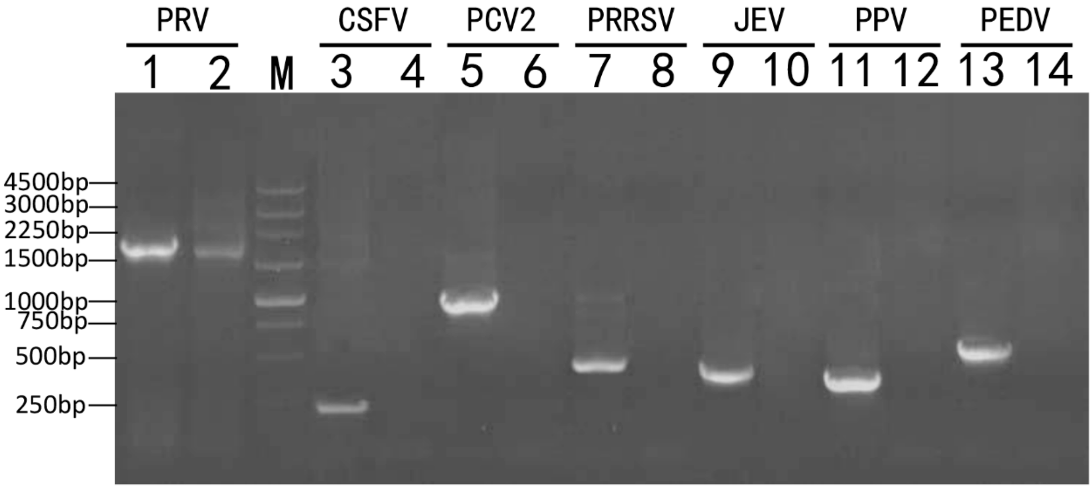

Figure S1. Identification of the cell cultures. M: 250bp Ladder DNA Marker; Line 1, 3, 5, 7, 9, 11 and 13 were the positive control for PRV, CSFV, PCV2, PRRSV, JEV, PPV and PEDV, respectively; Line 2 showed PRV positive of the cell culture, while line 4, 6, 8, 10, 12 and 14 showed negative for other pathogens.
